# “I Feel Sorry for Them”: Australian Meat Consumers’ Perceptions about Sheep and Beef Cattle Transportation

**DOI:** 10.3390/ani8100171

**Published:** 2018-10-03

**Authors:** Emily A. Buddle, Heather J. Bray, Rachel A. Ankeny

**Affiliations:** School of Humanities, University of Adelaide, Adelaide, SA 5005, Australia; heather.bray@adelaide.edu.au (H.J.B.); rachel.ankeny@adelaide.edu.au (R.A.A.)

**Keywords:** livestock, transport, live export, trucks, road transport, consumer perception, Australia

## Abstract

**Simple Summary:**

Understanding what concerns about animal welfare are most prominent among members of the public is critical to improve processes in the meat production industry. Hence, this study uses qualitative data to explore how Australian meat consumers viewed one aspect of the production process, livestock transportation. Participants in this study were concerned about the close packing of animals into trucks and ships, and their experiences during long-distance voyages; their views on this topic may be motivated by genuine concerns about animal welfare, together with anthropomorphic tendencies to project human feelings onto these animal experiences and emotional responses, due to transport being associated with slaughter. Given the importance of transport to the Australia red-meat production industry, we argue that public views should be considered as the sector modifies its practices; in addition, higher levels of transparency and communication about practices associated with good animal welfare are needed.

**Abstract:**

Concern for livestock welfare is significantly increasing in many parts of the world. One area of concern is the transportation of livestock. Using qualitative research methods, this research explores the concerns of Australian meat consumers related to livestock transportation practices, both on land by truck and on sea by ship. Participants were predominantly concerned about animals being “crammed” into trucks and ships, and the long distances over which livestock were transported. Likely contributors to these reactions are the high visibility of truck transport in urban areas, and recent media and political attention to the live-export issue in Australia. We argue that participants’ concerns about transport are arising for a variety of reasons, including anthropomorphic tendencies, genuine concern for the welfare of farm animals, and emotional responses related to the discomfort experienced by meat consumers when they are reminded of the meat-animal connection. Given the importance of transport to the red-meat production industry, these results suggest that the sector may need to reconsider some of their practices and increase transparency and communication about the practices, which they utilise to ensure good animal welfare.

## 1. Introduction

### 1.1. Context of the Study

Increasing public concern for the welfare of animals used for food is well documented in several countries [1,2,3], including Australia [4,5,6], and has been attributed to increased awareness of animal sentience [7] alongside a growth in intensification of agriculture and the increasing urbanisation of societies. Taylor and Signal [8] argue that urbanisation has led to a “progressively ‘removed’ form of knowledge regarding animals [which is] gained through, for example, nature programs and zoos rather than through exposure to primary production processes” [8] (p. 348). The data presented in this paper have been generated in the context of a project that aims to explore Australian meat consumers’ attitudes toward farm animal welfare in the Australian sheep and beef cattle industries. In qualitative research, participants sometimes take their responses into unexpected domains or emphasise particular issues much more than researchers might anticipate. In this study, we found that many concerns expressed by participants about red meat production focused on the transportation of livestock in trucks and also by sea as part of Australia’s live-export trade (discussed in more detail below). Hence, in this paper we specifically focus on concerns about livestock transport. We were particularly interested in whether participants spontaneously offered explanations of why they were concerned about transport and sought to have participants articulate their reasoning behind the explanations that they provided.

### 1.2. Human-Animal Relations and Livestock Production

Sociological research exploring human-animal relations has highlighted changes in public attitudes toward animals over the past 40 to 50 years, with an evolution from a traditional utilitarian perspective to a more compassionate and empathetic level of care. This change has been attributed to the growth of post-material values within Western societies more generally [9,10,11,12]. These changes in human-animal relations have resulted in increased tendencies to anthropomorphise, that is, to attribute human mental states (thoughts, feelings, motivations and beliefs) to non-human objects or creatures [13]. Many now view animals as moral others who are perceived to have distinct emotions and personalities [14]. Jasper and Nelkin [15] suggest that these changes in attitudes toward animals are a result of “sentimental anthropomorphism”, which stems from increasing urbanisation and a shift in focus from considering animals to be part of food production to viewing animals as increasingly meaningful members of society and/or the family. In the context of livestock production, when the characteristics of the production system are perceived to cause harm or suffering to animals, for example, because the free will of the animal is taken away, the specific conditions responsible for the suffering are likely to be perceived as immoral [16] and disgusting [17,18], particularly as people tend to anthropomorphise and consider how the animal is likely to be feeling in its current situation. As a result, many studies have demonstrated that people have become increasingly concerned about intensive animal agriculture, and that they prefer animals to be reared in free-range systems, which allow them to express “natural behaviours” [1,2,18,19].

### 1.3. Previous Work on Consumer Perceptions of Livestock Transport

Long distance transport of livestock is common in many countries, due to the low cost of transport relative to market value, market demand and relative seasonal variation, geographical concentration of production, and contract agreements [20,21]. Some researchers working in locales other than Australia have previously argued that people with little or no connection to agriculture lack knowledge about the practices associated with livestock transportation and animal welfare considerations during transport [22,23,24], and thus, many tend to believe that minimum to no transportation during an animal’s life is essential to good animal welfare [25]. Previous research from Europe has revealed that space allowance, shockproof and calm transportation methods, and duration of transport were of most concern to participants [1]. Similarly, long distances travelled to slaughter have also proved problematic for Canadians [2].

### 1.4. Livestock Transport in Australia

Australia is a large country and livestock may be required to be moved long distances to meat processors or to ports for export [21], and there are clear standards in Australia to help ensure animal welfare during transportation. Within the transportation guidelines as published by Animal Health Australia [26], it is indicated that animals should not be loaded too tightly or too loosely to minimise the risk of animal injury, and that internal trailer gates should be closed during transportation to ensure that the stocking density is evenly distributed within the trailer. The guidelines also state that cattle older than six months and sheep older than four months must not be off water for more than 48 h, which in turn generates the basis for the allowable travelling time.

One of the most visible parts of the sector where Australian livestock are subject to long distance transport is within the highly contentious live export trade [27], which is currently one of the most debated issues in association with livestock welfare in Australia. Debates over and campaigns against live export have been long-standing, resulting in the 2011 suspension of the cattle trade to Indonesia [28,29], and subsequent resumption of the trade, which received prominent coverage in the Australian media [28,30,31,32]. This media coverage also resulted in many people expressing outrage and campaigning against the trade on social media [33,34,35]. Research specifically into attitudes to the live export trade reveal that a majority of the Australian public does not support the trade [27,30].

### 1.5. Research Objectives and Approach

The study on which this paper is based endeavoured to explore the understandings of meat consumers about the welfare of sheep and beef cattle in Australia. While transportation of livestock is a major part of Australian livestock production, there is limited information about how Australian meat consumers feel about livestock transportation practices outside of the context of live export. Participants in our project were not asked explicitly about particular production practices; instead we used open-ended, general lines of questioning to form deeper understandings of what was of most concern to participants about farm animal welfare.

Our methods are based on the generic inductive qualitative model [36,37], which combines the data-gathering processes with description and interpretation while establishing research questions, along with purposeful sampling, including demographic-based recruitment. This approach strengthens our abilities to generalise, for example across populations and to other locations. However, this approach does not seek to generate statistically significant data or strict representativeness, due to the qualitative nature of the research methods utilised [37,38], which put greater emphasis on what participants say rather than how many participants made one particular claim.

In this paper, we use thematic analysis of interviews and focus groups to examine Australian meat consumers’ attitudes toward livestock transportation. Unlike in some previous qualitative studies (for example Clark et al. [39]), concerns associated with the transportation of livestock within the current research arose spontaneously. We contend that transport was of particular concern for our participants, due to the visibility of truck transport within the locations where interviews and focus groups were conducted. Livestock trucks are commonplace on major thoroughfares in Adelaide and Toowoomba, and to a lesser degree in Melbourne, which were our three locations for data gathering. In relation to livestock transportation by ship, the issue of live export has been an extremely contentious public issue in Australia, which has received significant media and political attention. Due to the open nature of questioning used in the current study, we were able to explore why transport was of particular concern for participants. Our findings suggest that consumers are concerned about animals being tightly packed or “crammed” into trucks and live export vessels and transported long distances particularly to slaughter, though there are several reasons that might underlie these concerns. We conclude that our findings suggest that the animal meat sector may need to reconsider some of their practices to align them more closely with community expectations along with current animal welfare science, and that they should increase transparency and communication about the practices, which they utilise to ensure good animal welfare, particularly those associated with transport.

## 2. Materials and Methods

This research was approved by the University of Adelaide’s Human Research Ethics committee (H-2012-262) and conducted according to Australian national guidelines [40]. Participants were required to be over the age of 18 and regular consumers of meat to participate in this research. Overall, 66 meat consumers from across Australia participated in the research during 2015 and 2016. Of these participants, 67% were women. Participants’ ages ranged from 18–24 years to over 65.

The research was conducted in Adelaide, South Australia (population of approximately 1.2 million), Melbourne, Victoria (population of approximately 4.65 million), and Toowoomba, Queensland (population of approximately 115,000). Focus groups and “mall-intercept” interviews [41] were used to collect data, with the latter utilised primarily to provide a balance in the overall sample in terms of demographics, particularly socioeconomic status. Three focus groups were conducted (2 in Adelaide, 1 in Melbourne) with 9 participants in each group (a total of 27 participants (40.9%)). The remaining 39 participants (59.1%) were involved in mall-intercept interviews. Focus group participants were recruited through community and social media announcements and flyers distributed at public events.

Focus groups and interviews were structured and included a series of discussion points about the welfare of sheep and beef cattle more broadly; however, this paper focuses on the concerns expressed by participants about the welfare of animals during transportation by truck and ship. Questions directed at participants were structured to remain open ended to allow them to express their own thoughts and use their own preferred concepts and language, rather than restricting their responses by providing a series of predetermined answers from which to select. The use of open-ended questions without prompted answers enabled us to develop a greater understanding about which issues were most important to the participants and also allowed them to reflect on their experiential knowledge [42]. Asking the same basic questions in each focus group and of each participant provided a foundation to aid comparison of results.

Focus groups and interviews were digitally recorded, and then transcribed fully, checked for accuracy against handwritten notes and anonymised. Each transcript was treated as a rich, narrative text and the first author inductively coded all open responses in NVivo [43] using methods similar to ‘open-coding’ [44]. These codes were subsequently reorganised, combined, and relabelled into a smaller number of general themes through a process of constant comparison. The final themes were later organised under overarching categories, and illustrative examples selected for reporting the findings. Measures of trustworthiness and rigour included comparison of coding of a subset of transcripts with the second author and discussion of all coding and themes with all authors. Quotes used in the results and discussion are illustrative and are typical of those coded to the themes identified during analysis.

## 3. Results

In this section, we briefly present evidence from our data based on participants’ responses, organised by key themes. We analyse these data in more detail in the discussion.

### 3.1. Transport as Compared to Other Concerns

As mentioned previously, participant responses to open questions about areas of concern within the red meat production sector highlighted that transport was a key area that was considered to impact negatively on farm animal welfare. This concern was particularly apparent in the context of the responses to the initial orientation question, which asked participants to describe the images that come to mind when they think about beef cattle or meat sheep farming, as illustrated below:

Researcher: So when I ask you to get a picture in your mind about animal farming, beef or sheep cattle what kind of images come to your mind?

US3: Umm what disturbs me is animal transport

Researcher: Right

US3: So I just see animals packed into vehicles

Researcher: So that is what you see here in the streets of Adelaide or are you talking about what you see on television?

US3: Uh no what I see because I live at Crafers [A suburb in the foothills east of Adelaide and connected to the Central Business District (downtown) by a freeway that connects the city with farmland and a large abattoir] and I am up and down the freeway regularly so we pass next to or behind the vehicle like that. I do find it quite distressing.

Researcher: When you think of cattle farming in your head, what kind of images do you see?

PFG8: I think of umm the sheep in the uhh trucks, all shoved in there, that’s what I think of yeah. On Portrush Road [This road is a major north-south thoroughfare in Adelaide that also runs through high SES suburbs] yeah.

Researcher: So when I say sheep and beef farming, what do you think of? What sorts of images come to mind?

SB4: Oh, crowded cattle trucks, you know, animals being badly bloody treated actually.

Researcher: Can you elaborate a bit more on that?

SB4: You know, you see some cattle trucks sometimes, they’re absolutely, animals crammed in, you know? You see the live beef export, that’s disgusting. You see the ships being loaded up with sheep and that, I don’t know if they still do live sheep exports. And I’ve seen some of that … documentary, I forget what they call it now but it showed film, belting the crap out of them so yeah I just think of cruelty.

Researcher: When I talk about sheep and beef farming, what sort of pictures come to your head?

GB1: Sheep and beef farming. Well I see the sheep out in the paddock [pause] … I don’t like the way they get pushed into the trucks.

Researcher: You don’t like the trucks?

GB1: No. They’re pushed in, they push, push, just how many get in? Then I, what I don’t like when they get transported.

Themes emerging from the data reveal that the participants in this research described transport in negative terms, such as “cruelty”, and used emotional language, such as “distress” and “disgust”, to express their perceptions of transport that they both witnessed first-hand, as well as via media representations. In summary, participants perceived that various aspects of livestock transportation had a negative impact on animal welfare and were concerned. As there were notable differences in the way participants described transport via truck and transport via ship, the following sections examine these modes of transport separately.

### 3.2. Perceptions of Transport via Truck

As seen in the quotes above, transport by truck was described in emotional language by participants. The predominant concern was the idea that animals are “crammed”, “shoved”, or forced into trucks, with limited space to move, and that this was “bad treatment”.

Other participants described transport in terms of the emotions expressed by the animals being transported, as illustrated below:

US2: It really troubles me on a really hot day when you drive down the road and there is a cattle van or a chicken truck and it’s a really hot day and they are in a metal van and they’re all just crammed in against the hot walls … they look troubled and you know you see them sniffing at the side of the thing and it’s horrible. It makes me really, really sad.

This quote also illustrates participants’ concerns about the impacts of exposure to the environment on farm animal welfare during transportation. In addition, participants perceived that there were risks of injury during transportation:

GB1: …[I’m] not happy when they get pushed into trucks, especially up I think in the Northern Territory well where there’s big trucks and … sometimes the leg hangs out and I don’t like the way they get transported.

Participants were also concerned about the impacts of long-distance transportation on animal welfare and linked these impacts to the resulting meat quality:

Researcher: You mentioned ah slaughter … are there any aspects of that that concern you more than others?

MFG9: Ah yeah, quite a lot, particularly in Victoria because we have very structured system which makes it very difficult for farmers to, for instance, go to a smaller abattoir. A lot of time they are forced to take the animals long distances which often means putting them into, jamming them into trucks and putting the animals under an enormous amount of stress umm which I think is bad for the animals, it is bad for the meat.

In summary, participants expressed concerns about animals being tightly packed for transportation via truck and associated transportation with exposure to the environment and risk of injury, both of which they viewed as impacting negatively on animal welfare.

### 3.3. Perceptions of Transport via Ship

Similar to perceptions of truck transport, participants expressed disgust and concern for animal suffering when animals were tightly packed on live export vessels and transported long distances:

Researcher: You don’t like live export?

UB1: Not really. Have you ever been on one of the ships? It is absolutely foul.

Researcher: Are you worried about the animals on the [live export] trip or about what happens to them when they get to the other end?

SS2: Both because you know they’re packed so tightly and haven’t got water and access to. I don’t know. I just know it’s a long journey and it’s cruelty and they suffer.

Participants were not only worried about the transportation process, but also expressed concerns about the destination countries, often voicing fears about how the animals are treated in countries who receive livestock through the live export supply chain. Concerns about animal treatment in receiving countries were generally associated by participants with halal slaughter practices as most countries which receive live animals from Australia have a Islamic majority.

Researcher: Are there any issues you are unsure about associated with beef and sheep production?

SB2: I am only unsure of when the sheep or livestock is shipped overseas. To say for instance to Indonesia or any Asian country or anywhere, how they are treated there. I have got a bit of a thing about that because there has been programs on TV … [talking about] the inhumane attitude they have toward these animals. So it is a concern, I must admit.

Many participants advocated for the live export industry to be closed, often suggesting that processing in Australia would be a better option.

UB3: I feel that the live animal export is a major issue as far as I am concerned personally and everyone in my circles. I think we can do far more for this country by bringing onshore the abattoirs and processing situations and if [other] countries insist on having our live animals then I am afraid it should be a closed door and we seek other markets that do accept our processed meat.

Ultimately, participants believed the live export trade to be unnecessary and often questioned why animals are subject to poor conditions on ships when in their views other viable alternatives exist within the Australian meat processing sector.

### 3.4. Transporters as the Problematic ‘Middle Men’

Participants’ perceptions revealed that they viewed transportation as a distinct stage in the production of red meat and one that was beyond the ‘care’ of farmers. Transporters were viewed as ‘middle men’, and were considered to include those working in the parts of the value chain between farmers and consumers, those involved in the sale of livestock in saleyards, and arguably even those in the retail sector as revealed in the quotes below:

Researcher: Do you think farmers do enough to ensure good animal welfare?

UB3: Umm we are talking about sheep and cattle, yes. But it is after that when they go out to the live trade umm that worries me. In the trucks, that worries me. Once they leave the farm gate I am concerned. The third, the second, the person in the middle. The middle man before the abattoir I really care about…

SB2: That is what it is all about. It’s all a business. It is the second and third man that make [agricultural] business a problem.

### 3.5. Transport to Slaughter

The fact that animals in livestock trucks were being transported to slaughter also impacted on participants’ perceptions of transportation. Participants expressed that there is a special responsibility on the part of transporters and others to ensure good welfare because animals were travelling to “their deaths”, as exemplified by the following quotes:

SS3: Umm I just think of the animals and I feel sorry for them because ultimately they are going to be killed and that’s, what, emotionally I think ohh that isn’t good.

US2: I think the way that they are transported from their farm or their shed or wherever they are coming from to go to the place they are killed, some thought needs to be put into that as well … So I feel like they’re, if it could be just one bad day, I guess that’s nice but making sure that day is not painful and unpleasant as possible.

These quotes demonstrate that participants respond emotionally to the idea of animals travelling to slaughter and believe that animals should be treated particularly well during this process.

## 4. Discussion: Why is Transport So Problematic?

### 4.1. Visibility

Transport was a key area of concern when participants described their perceptions of red meat consumption. Transport may be of greater concern to the general public than other practices affecting farm animal welfare, for example painful practices in animal care and production, because transport is highly visible to the public, due to livestock trucks passing regularly through urban areas. Participants (in Adelaide and Toowoomba in particular) mentioned seeing trucks in their neighbourhoods and on main roads. For many in urban areas, the only occasion on which they see meat producing animals may well be during transport. Areas of beef cattle and sheep meat production are often remote from urban areas, but in smaller cities the rationalisation of the meat processing sector means that animals may need to be transported from one side of the city to the other, with the shortest route being through urban areas.

In addition to the first-hand observations of animals in trucks in urban areas, the live export industry has frequently been covered in the Australia press in recent years [45,46] following on from exposés revealing abuse in destination countries, appalling conditions during transport, and continued breaches of industry standards. It should be noted that we are not suggesting that the media coverage of live export is influencing public perceptions more generally of transportation, only that the issue of animal transport has been prominent in public discourse in Australia in recent years via the live export debates. The perceptions of live export expressed by the participants in this study are consistent with those documented in other scholarship [27,30] and include “disgust”, outrage, and calls for the trade to be discontinued, as we discuss in more detail below.

### 4.2. The Role of Anthropomorphism

Concerns expressed in the current study about animals being tightly packed into trucks or ships may result from participants projecting their emotions onto the animals based on the participants’ own experiences in crowded situations. Crowding is a subjective psychological response to density and refers to situations which result in feelings of restriction within the individuals exposed to limited or tight spaces [47,48]. In relation to human population density, different cultures have different levels of adaptation and tolerance to crowding [48]. Tolerance of crowding is related to socioeconomic background, age, education, culture, and previous living environments. People who have previously lived in a high density, crowded environment are less likely to feel crowded than someone who lives in a more spacious environment [48,49,50]. In Australia, we have relatively low population density relative to other countries; however, Australians are still likely to have experienced situations of crowding, such as at a sporting event or music concert and thus can still imagine discomfort at being crowded or “crammed” into a small space. Long distance transport could be viewed in the same way: Many people do not like to travel in tight spaces, such as planes or cars, for long periods or distances. In short, concern about the density at which animals are loaded for transportation may be reflections of the participants’ own feelings about crowding and confinement.

### 4.3. What about Disgust and Moral Outrage?

Participants frequently described their perceptions of livestock transport as ‘disgusting’. Several authors have suggested links between disgust and moral outrage [51,52,53,54]. Disgust is considered to be a moral emotion [55] and a “gut feeling” [56], which is provoked by something, or the thought of something, violating the purity of the body or soul [57]. The feeling of disgust has previously been identified as resulting from imagery of animals raised under ‘cruel’ factory farming conditions [17,18]. Some authors suggest that people find things more immoral when they are exposed to disgust cues (such as the presence of contaminants, for example faeces) [56]. Therefore, it may be that the current study’s participants found what they viewed as the crowded and potentially unsanitary conditions experienced by animals during transport to be disgusting and hence morally problematic. This disgust may also be connected to the idea that these animals will eventually be consumed, as disgust often arises in connection to issues relating to food consumption [58,59,60]. Alternatively, participants may have found the treatment of animals during transport to be morally problematic (because it is “cruel”) and hence “disgusting”. It is difficult to disentangle these concepts or to definitively determine the causality between them, given their tight relationship in our participants’ responses.

### 4.4. The Connection of Transport to Slaughter

The visibility of animals in trucks (or on ships in the media or in person) may serve as a reminder that animals are killed to produce meat and hence may reinforce the meat-animal connection, which may be distressing even for meat consumers. In Australia, meat is typically purchased from the supermarket or a butcher’s shop in pre-packaged forms and generally lacks much resemblance to the live animal that it once was [61,62]. Disguising the animal characteristics of meat by removing prompts, such as the head, feet, and skin, removes the associated personality and intelligence of the living animal, which further enables the de-animalising process [63]. Supermarkets have contributed to this process by presenting ready-cooked meat in packages, which makes the original animal even more distant from products with uncooked animal flesh, which arguably has more transparency with regard to its origins [64]. The distinction between the animal and meat produced from it is further complicated through creation of semantic differences particularly in English, which eliminates animal designators, such as cow and sheep, by substituting terms, such as beef and lamb in the context of consumption [63]. Such differentiation reinforces the de-animalising process, which allows consumers to generate mental distinctions between the living animal and meat product, and hence detach the one from the other. As Leroy and Praet [65] describe, even with an increasing level of moral aversion toward animal killing, those in modern societies are still fond of consuming meat, though they are experiencing increasing levels of ambivalence.

### 4.5. Genuine Concern for the Welfare of Animals

Negative emotions toward livestock transport highlighted in the current study appear to be related to animals being “crammed” into trailers or ships, which participants felt was cruel and inhumane. Australia has one of the highest rates of pet ownership globally, with 62 percent of households owning pets [66]. Many Australian families have become “more-than-human” [67] with a higher proportion of Australians living with a dog and/or cat than with a child [68]. Alongside the increase in pet ownership, there has been an incredible economic rise in the pet industry, with Australian households spending more than $12.2 billion annually on pet products and services, representing an increase of 42 percent from 2013 to 2016 [69].

Australians’ increasing concerns about farm animal welfare can also be seen in the response to recent activists’ campaigns about caged hens [70] and the live export issue [17,28]. In activist campaign videos, animals are often represented as individuals (frequently with names and personalities) who are suffering. The activist organisation Animals Australia has been at the forefront of broadcasting moments of “extreme cruelty experienced by these animals in their last moments” in both domestic and overseas abattoirs with such footage described as “effective enough in mobilising Australians for abattoirs to be shut down…and the installation of surveillance cameras in abattoirs” [17] (p. 45), as well as the suspension of live export trade [28]. Despite evidence which suggests that Australian meat consumers typically ignore animal welfare activism, particularly when it is online [38], it is clear that animal welfare activism has played a significant role in raising general awareness and communicating to the public about intensive animal agriculture and the live export controversy.

In the case of live export in particular, many Australians wish to see the trade end [27]. As live export has been a long-standing and ongoing point of contention in Australia, it is unsurprising that it was raised as an issue associated with animal welfare by participants in the current study. Many participants stated that the trade is disgusting and unnecessary, as processing the livestock in Australia and exporting the frozen meat was considered to be more likely to be beneficial to the Australian workforce and economy than are current practices associated exporting the live animal. However, it is unclear whether such an approach would be viable, given that Australia’s main export markets require halal slaughter and limited time between slaughter and consumption, and also often do not have extensive refrigeration networks.

### 4.6. Transport Workers are Not Trusted

Although farmers are generally trusted in Australia to care for the welfare of their animals [71,72], this trust is not extended to those who transport animals. Coleman et al. [73] have demonstrated that 24 percent of the general public in Australia have low trust in workers involved in land-based livestock transport, and 41 percent indicated to have low trust in workers involved in livestock transport by sea. Similar results were found in the current study, with participants often suggesting those involved between the farm gate and the processing plant (i.e., the livestock transporters) are of most concern. Given these public concerns, the industry may need to consider ways to limit or eliminate long distance truck transport by opening, or in the case of Australia re-opening, smaller more localised processing facilities or utilising mobile abattoirs [2]. However, these options may be unrealistic based on the efficiencies obtained by utilising larger processing plants, as well as the high cost of livestock processing in Australia.

## 5. Conclusions

This paper documents qualitative data that indicates that livestock transport is a key area of concern for Australian meat consumers and is viewed as having negative impacts on animal welfare. The spontaneous responses provided by participants indicate that transport is more prominent among the concerns of meat consumers than other practices that may impact negatively on animal welfare, such as painful procedures. Participants used emotive language to describe their perceptions of transport, including “disgust” and “sadness”, and the idea that animals were treated “cruelly” during transport. Negative associations with animals being closely packed together during transport was a strong theme that emerged from the data. Animals themselves were also described as appearing “stressed” and “troubled” during transport. We contend that the high visibility of truck transport in urban areas, and the media and political attention given to the live-export issue in Australia, are likely contributors to the prominence of transport as an area of concern. We also suggest that this concern may be arising for a variety of reasons, including, due to anthropomorphic tendencies, genuine concern for the welfare of farm animals, and/or emotional responses related to the discomfort experienced by meat consumers when they are reminded of the meat-animal connection, given that animals being transported are on their way to a slaughter facility.

The evidence from our work and research by others [19] suggests that the livestock transport sector has to some extent lost consumer trust. We did not anticipate that transport would be as prominent in our research as it in fact was, nor did we expect to document the high visibility of livestock transport trucks in urban areas as described by our participants. Given the importance of transport to the red-meat production industry, these results suggest that the sector may need to reconsider some of their practices so that they are more in line with community expectations and with current animal welfare science, and to increase transparency and communication about the practices, which they utilise to ensure good animal welfare.

## References

[1] Vanhonacker F., Van Poucke E., Tuyttens F., Verbeke W. (2010). Citizens’ views on farm animal welfare and related information provision: Exploratory insights from Flanders, Belgium. J. Agric. Environ. Ethics.

[2] Spooner J., Schuppli C., Fraser D. (2014). Attitudes of Canadian citizens toward farm animal welfare: A qualitative study. Livest. Sci..

[3] Gracia A. (2013). The determinants of the intention to purchase animal welfare-friendly meat products in Spain. Anim. Welf..

[4] Coleman G.J., Rohlf V., Toukhsati S., Blache D. (2015). Public attitudes relevant to livestock animal welfare policy. Farm Policy J..

[5] Doughty A.K., Coleman G.J., Hinch G.N., Doyle R.E. (2017). Stakeholder perceptions of welfare issues and indicators in extensively managed sheep in Australia. Animals.

[6] Bray H.J., Buddle E.A., Ankeny R.A. (2017). What are thinking? Consumer attitudes to meat production in Australia. Anim. Prod. Sci..

[7] Hötzel M.J., Appleby M.C., Weary D.M., Sandøe P. (2014). Improving farm animal welfare: Is evolution or revolution needed in production systems?. Dilemmas in Animal Welfare.

[8] Taylor N., Signal T.D. (2009). Willingness to Pay: Australian Consumers and “On the Farm” Welfare. J. App. Anim. Welf. Sci..

[9] Franklin A., White R. (2001). Animals and modernity: Chaining human-animal relations. J. Sociol..

[10] Mazur N., Maller C., Aslin H., Kancans R. (2006). Australian Animal Welfare Strategy Stakeholder Analysis Phases 1-4.

[11] Goodfellow J., Tensen M., Bradshaw L. (2014). The future of animal welfare policy and its implications for Australian livestock industries. Farm Policy J..

[12] Goodfellow J., Cao D., White S. (2016). Regulatory capture and the welfare of farm animals in Australia. Animal Law and Welfare–International Perspectives.

[13] Serpell J.A., Datson L., Mitman G. (2005). People in disguise: Anthropomorphism and the human-pet relationship. Thinking with Animals: New Perspectives on Anthropomorphism.

[14] Fessler L. People Who Talk to Pets, Plants, and Care are Actually Totally Normal, According to Science. https://qz.com/935832/why-do-people-name-their-plants-cars-ships-and-guitars-anthropomorphism-may-actually-signal-social-intelligence/.

[15] Jasper J.M., Nelkin D. (1992). The Animal Rights Crusade: The Growth of a Moral Protest.

[16] Gray K., Young L., Waytz A. (2012). Mind perception is the essence of morality. Psychol. Inq..

[17] Mummery J., Rodan D. (2017). Mediation for affect: Coming to care about factory-farmed animals. Media Int. Aust..

[18] Bray H.J., Ankeny R.A. (2017). “Happier chickens lay tastier eggs”: Motivations for buying free-range eggs in Australia. Anthrozoös.

[19] Coleman G., Jongman E., Greenfield L., Hemsworth P. (2016). Farmer and public attitudes toward lamb finishing systems. J. Appl. Anim. Welf. Sci..

[20] Carlsson F., Frykblom P., Lagerkvist C.J. (2007). Consumer willingness to pay for farm animal welfare: Mobile abattoirs versus transportation to slaughter. Eur. Rev. Agric. Econ..

[21] Coleman G.J. (2018). Ublic animal welfare discussions and outlooks in Australia. Anim. Front..

[22] Te Velde H., Aarts N., Van Woerkum C. (2002). Dealing with ambivalence: Farmers’ and Consumers’ perceptions of animal welfare in livestock breeding. J. Agric. Environ. Ethic..

[23] Vanhonacker F., Verbeke W., Van Poucke E., Tuyttens F.A.M. (2008). Do citizens and farmers interpret the concept of farm animal welfare differently?. Livest. Sci..

[24] Vanhonacker F., Verbeke W. (2014). Public and consumer policies for higher welfare food products; Challenges and opportunities. J. Agric. Environ. Ethic..

[25] Miele M., Evans A., Butterworth A. (2005). European consumers view about farm animal welfare. Welfare Quality Conference: Science and Society Improving Animal Welfare.

[26] Animal Health Australia Australian Animal Welfare Standards and Guidelines–Land Transport of Livestock. http://www.animalwelfarestandards.net.au/files/2015/12/Land-transport-of-livestock-Standards-and-Guidelines-Version-1.-1-21-September-2012.pdf.

[27] Sinclair M., Derkley T., Fryer C., Phillips C.J.C. (2018). Australian public opinions regarding the live export trade before and after an animal welfare media expose. Animals.

[28] Munro L. (2014). The live animal export controversy in Australia: A moral crusade made for the mass media. Soc. Mov. Studies: J. Soc. Cult. Pol. Protest.

[29] Petrie C. Live Export: A Chronology. https://www.aph.gov.au/About_Parliament/Parliamentary_Departments/Parliamentary_Library/pubs/rp/rp1617/Chronology/LiveExport.

[30] Tiplady C.M., Walsh D.-A.B., Phillips C.J.C. (2013). Public response to media coverage of animal welfare. J. Agric. Environ. Ethic..

[31] Fozdar F., Spittles B. (2014). Of cows and men: Nationalism and Australian cow making. Aust. J. Anthro..

[32] Rolls M. (2017). Casuistry, commentary and killing cattle: Transgressing notional borders of belonging. J. Aust. Studies.

[33] Schoenmaker S., Alexander D. (2012). Live cattle trade- the case of an online crisis. Soc. Altern..

[34] Rikken M. (2013). Campaigning in a changing media environment: The Public as a Creator, Consumer and Distributor of Information. Master’s Thesis.

[35] Buddle E.A., Bray H.J., Pitchford W.S. (2017). Keeping it “inside the fence”: An examination of responses to a farm animal welfare issue on Twitter. Anim. Prod. Sci..

[36] Maxwell J.A. (2005). Qualitative Research Design: An Interactive Approach.

[37] Hood J.C., Bryant A., Charmaz K. (2007). Orthodoxy vs. power: the defining traits of grounded theory. The SAGE Handbook of Grounded Theory.

[38] Buddle E.A., Bray H.J., Ankeny R.A. (2018). Why would we believe them? Meat consumers’ reactions to online farm animal welfare activism in Australia. Commun. Res. Pract..

[39] Clark B., Stewart G.B., Panzone L.A., Kyriazakis I., Frewer L.J. (2016). A systematic review of public attitudes, perceptions and behaviours towards production diseases associated with farm animal welfare. J. Agric. Environ. Ethic..

[40] National Health and Medical Research Council, Australian Research Council, The Australian Vice-Chancellors’ Committee National Statement on Ethical Conduct in Human Research, Updated July 2018. https://www.nhmrc.gov.au/guidelines-publications/e72.

[41] Bush A.J., Hair J.F. (1985). An assessment of mall intercept as a data collection method. J. Mark. Res..

[42] Denzin N.K., Lincoln Y.A. (2011). The Sage Handbook of Qualitative Research.

[43] Richards L. (2005). Handling Qualitative Data: A practical Guide.

[44] Strauss A., Corbin J. (1990). Social Sciences–Statistical Methods; Grounded Theory; Qualitative Research.

[45] Four Corners (2011). A Bloody Business.

[46] Calcutt L., Little L. 60 Minutes Uncovers Disturbing Video from Live Sheep Export Vessel. https://www.9news.com.au/national/2018/04/08/21/06/60-minutes-live-export-sheep-vessel.

[47] Stokols D. (1972). A social-psychological model of human crowding phenomena. Am. Inst. Plann. J..

[48] Yeh A.G.O., Yeh A.G.O., Ng M.K. (2000). The planning and management of a better high density environment. Planning for a Better Urban Living Environment in Asia.

[49] Anderson E.N. (1972). Some Chinese methods of dealing with crowding. Urban. Anthro..

[50] Schmidt D., Goldman R., Feimer N. (1976). Physical and psychological factors associated with perceptions of crowding: An analysis of subcultural differences. J. Appl. Psych..

[51] Darley J.M., Pittman T.S. (2003). The psychology of compensatory and retributive justice. Pers. Soc. Psychol. Rev..

[52] Mullen E., Skitka L.J. (2006). Exploring the psychological underpinnings of the moral mandate effect: Motivated reasoning, group differentiation, or anger?. J. Pers. Soc. Psychol..

[53] Jensen N.H., Petersen M.B. (2011). To defer or to stand up? How offender formidability affects third party moral outrage. Evol. Psychol..

[54] Salerno J.M., Peter-Hagene L.C. (2013). The interactive effect of anger and disgust on moral outrage and judgements. Psychol. Sci..

[55] Rozin P., Lowery L., Imafa S., Haidt J. (1999). The CAD triad hypothesis: A mapping between three moral emotions (contempt, anger, disgust) and three moral codes (community, autonomy, divinity). J. Pers. Soc. Psych..

[56] Schnall S., Haidt J., Clore G.L., Jordan A.H. (2008). Disgust as embodied moral judgment. Pers. Soc. Psychol. Bull..

[57] Horberg E.J., Oveis C., Keltner D., Cohen A.B. (2009). Disgust and the moralization of purity. J. Pers. Soc. Psych..

[58] Rozin P., Millman L., Nemeroff C. (1986). Operation of the laws of sympathetic magic in disgust and other domains. J. Pers. Soc. Psych..

[59] Clifford S., Wendell D.G. (2016). How disgust influences health purity attitudes. Polit. Behav..

[60] Shriver A., McConnachie E. (2018). Genetically modifying livestock for improved welfare: A path forward. J. Agric. Environ. Ethic..

[61] Bulliet R. (2005). Hunters, herders and hamburgers. The Past and Future of Human-Animal Relationships.

[62] Leroy F., Praet I. (2015). Meat traditions: The co-evolution of humans and meat. Appetite.

[63] Plous S. (1993). Psychological mechanisms in the human use of animals. J. Soc. Issues.

[64] Hoogland C., de Boer J., Boersema J. (2005). Transparency of the meat chain in the light of food culture and history. Appetite.

[65] Leroy F., Praet I. (2017). Animal killing and postdomestic meat production. J. Agric. Environ. Ethic..

[66] RSPCA How Many Pets are there in Australia?. http://kb.rspca.org.au/how-many-pets-are-there-in-australia_58.html.

[67] Irvine L., Cilia L. (2017). Teaching and learning guide for more-than-human families: Pets, people and practices in multispecies households. Soc. Compass.

[68] Roy Morgan Doggone it: Pet Ownership in Australia. http://www.roymorgan.com/findings/6272-pet-ownership-in-australia-201506032349.

[69] Australian Veterinary Association Pet Ownership Statistics. http://www.ava.com.au/news/media-centre/hot-topics-4.

[70] Rodan D., Mummery J. (2014). The ‘Make It Possible’ Multimedia Campaign: Generating a New ‘Everyday’ in Animal Welfare. Media Int. Aust..

[71] Cockfield G., Botterill L.C. (2012). Signs of countrymindedness: A survey of attitudes to rural industries and people. Aust. J. Polit. Sci..

[72] Worsley A., Wang W., Ridley S. (2015). Australian adults knowledge of Australian agriculture. Br. Food J..

[73] Coleman G.J., Toukhsati S., Rohlf V., Blache D. (2014). Development of a Public Attitude Monitoring Scheme APL Project Number 2012/0026 Final Report.

